# Maternal near-miss in a rural hospital in Sudan

**DOI:** 10.1186/1471-2393-11-48

**Published:** 2011-06-29

**Authors:** AbdelAziem A Ali, Awadia Khojali, Amira Okud, Gamal K Adam, Ishag Adam

**Affiliations:** 1 Department of Obstetrics and Gynecology, Faculty of Medicine, Kassala University, Kassala, Sudan; 2 Department of Obstetrics and Gynecology, Faculty of Medicine, Gadarif University, Gadarif, Sudan; 3 Department of Obstetrics and Gynecology, Faculty of Medicine University of Khartoum, Khartoum, Sudan

## Abstract

**Background:**

Investigation of maternal near-miss is a useful complement to the investigation of maternal mortality with the aim of meeting the United Nations' fifth Millennium Development Goal. The present study was conducted to investigate the frequency of near-miss events, to calculate the mortality index for each event and to compare the socio-demographic and obstetrical data (age, parity, gestational age, education and antenatal care) of the near-miss cases with maternal deaths.

**Methods:**

Near-miss cases and events (hemorrhage, infection, hypertensive disorders, anemia and dystocia), maternal deaths and their causes were retrospectively reviewed and the mortality index for each event was calculated in Kassala Hospital, eastern Sudan over a 2-year period, from January 2008 to December 2010. Disease-specific criteria were applied for these events.

**Results:**

There were 9578 deliveries, 205 near-miss cases, 228 near-miss events and 40 maternal deaths. Maternal near-miss and maternal mortality ratio were 22.1/1000 live births and 432/100 000 live births, respectively. Hemorrhage accounted for the most common event (40.8%), followed by infection (21.5%), hypertensive disorders (18.0%), anemia (11.8%) and dystocia (7.9%). The mortality index were 22.2%, 10.0%, 10.0%, 8.8% and 2.4% for infection, dystocia, anemia, hemorrhage and hypertensive disorders, respectively.

**Conclusion:**

There is a high frequency of maternal morbidity and mortality at the level of this facility. Therefore maternal health policy needs to be concerned not only with averting the loss of life, but also with preventing or ameliorating maternal-near miss events (hemorrhage, infections, hypertension and anemia) at all care levels including primary level.

## Background

Maternal near-miss cases of women who nearly died but survived a complication during pregnancy, childbirth or postpartum (maternal near miss or severe acute maternal morbidity) are increasingly recognized as useful means to examine quality of obstetric care since pregnancy complications occur in 15% of women worldwide. The practical implementation of maternal near miss concept should provide an important contribution to improving quality of obstetric care to reduce maternal deaths and improve maternal health [1-4]. More than half a million women die annually as a result of pregnancy. Therefore, it is important to investigate the causes of maternal deaths and maternal morbidities to reduce the maternal mortality ratio and thus meet the Fifth Millennium Development Goal set by the United Nations [5,6]. In the developing countries the unacceptably high maternal mortality overshadows severe maternal morbidity. However, near-miss events occur more frequently than maternal deaths. Therefore, more detailed and comprehensive studies on maternal near-miss have been conducted and are of value to clinical audit and practice [6-8].

Although a high maternal mortality ratio (644/100 000 live birth), with septicemia and hemorrhage as the main direct causes, was previously reported in Kassala hospital in eastern Sudan [9], there are no data on severe maternal morbidity and near-miss events. Thus, the present study was conducted to investigate the frequency of near-miss events, to calculate the mortality index for each event and to compare the socio-demographic data (age, parity, gestational age, education and antenatal care) of the near-miss cases with maternal deaths. These basic fundamental data will be valuable for supporting program managers in developing countries with evidence-base data.

## Methods

### Setting

Medical files of pregnant women and who delivered recently at Kassala Maternity Hospital from January 2008 to December 2010 were retrospectively retrieved. Kassala is located in eastern Sudan, 600 km from Khartoum; it is 42,282 square km, with a population of 1.8 million people. Of these, 440,491 women are of reproductive age. Kassala Hospital provides tertiary care for women who receive antenatal care at the hospital, as well as for referrals from other clinics and hospitals, and for women who live close to the hospital facility. All women with risk factors or obstetric complications are referred to the hospital. However, the referral criteria are not strictly adhered to and many patients without any significant complications deliver at the hospital.

### Participants

Maternal near-miss cases were defined as women with at least one near-miss event as follows: acute obstetric complications that immediately threaten a woman's survival but do not result in her death, either by chance or because of hospital care she receives during pregnancy, labor or within 6 weeks after termination of pregnancy or delivery [10]. For these events, the following disease-specific criteria that were employed by Filippi et al., [10] were applied: (I)hemorrhage leading to shock; emergency hysterectomy; coagulation defects and/or blood transfusion of ≥ 2 liters; (II)hypertensive disorders in pregnancy, including both eclampsia and severe pre-eclampsia with clinical/laboratory indications for termination of pregnancy to save the woman's life; (III)dystocia; uterine rupture and impending rupture, e.g., prolonged obstructed labor with previous caesarean section; (IV) infection with hyperthermia or hypothermia or a clear source of infection; and clinical signs of septic shock and (V) severe anemia (hemoglobin level <7 g/dl) [5,10].

### Data collection and analysis

For each obstetric condition, we calculated the mortality index to determine the standard of care provided for each disease process. The mortality index was expressed as a percentage and calculated as the number of maternal deaths resulting from a particular obstetric condition divided by the sum of the near-miss morbidities and maternal deaths resulting from such obstetric conditions. These near-miss cases were retrospectively retrieved among all pregnant and recently delivered women with pregnancy-related complications and who were admitted to the hospital. The data of cases and maternal deaths were gathered applying the above-mentioned criteria for definition of near-miss cases, and maternal death was defined as the death of a woman while pregnant or within 42 days of termination of pregnancy, irrespective of the duration and site of the pregnancy, from any cause. For each case, information on socio-demographic characteristics, parity, gestational age at the time of the near-miss morbidity, nature of the obstetric complication(s), presence of organ and/or system dysfunction, duration of hospital stay, and source of referral were collected. Socio-demographic data, parity and gestational age were compared between the near-miss cases and maternal deaths.

Data were entered in a computer database using SPSS (SPSS Inc., Chicago, IL, USA, version 16.0) for Windows and were double-checked before analysis. The Student's t-test and x^2 ^test were used to compare means and proportions, respectively. *P *< 0.05 was considered significant.

### Ethics

The research approved and received ethical clearance from the Health Research Committee in the ministry of health at Kassala State, eastern Sudan.

## Results

During the study period, there were 9578 deliveries, 9262 live births, 205 near-miss cases and 40 maternal deaths. A total of 228 near-miss events were identified among the near-miss cases, which implies that 23 women had more than one event, yielding a mean of 1.1 near-miss morbidities per case. This is resulted in a total maternal near-miss and maternal mortality ratio of 22.1/1000 live births and 432/100 000 live births, respectively. The total mortality index for near-miss cases was 19.5% (near-miss/fatality ratio 1:5.1). Hemorrhage accounted for the most common event (40.8%), followed by infection (21.5%), hypertensive disorders (18.0%), anemia (11.8%) and dystocia (7.9%), table 1.

**Table 1:** Identified near-miss events in Kassala hospital, Sudan 2008 - 2010

Near-Miss event	Number	Percentage of the total
Hemorrhage	93	40.8
Ectopic	10	4.4
Abortion	4	1.8
Placenta previa	8	3.5
Abruptio placentae	18	7.9
Postpartum hemorrohage	53	23.2
Other	00	00
Hypertensive disorders	41	18
Pre-eclampsia	28	12.3
Eclampsia	13	5.7
Dystocia	18	7.9
Impending rupture	13	5.7
Ruptured uterus	5	2.2
Infection	49	21.5
Anemia	27	11.8
Total	228	100

There were no cases reported of organ or system dysfunction/failure. The duration of hospital stay ranged from 3 to 15 days. Most of the cases (42.9%) were referred from surrounding rural hospitals.

During the period of the study there were 316 (33/1000 birth) and 86 (9/1000 live birth) stillbirths and neonatal deaths, respectively. Likewise, 23.7% and 5.9% stillbirths and early neonatal deaths respectively were observed among 152 women of the near-miss cases that associated with labor.

Various causes of maternal deaths were identified, table 2. The mortality index was 22.2% 10.0%, 10.0%, 08.8% and 02.4% for infection, dystocia, anemia, hemorrhage and hypertensive disorders, respectively, table 3.

**Table 2:** Cause of maternal mortality in Kassala hospital, Sudan: 2008-2010

Factor	Maternal death (*n *= 40)	Percentage of the total
Septicemia/infections	14	35
Hemorrhage	9	22.5
Embolism	4	10
Malaria	4	10
Anemia	3	7.5
Ruptured uterus	2	5
Jaundice of unknown cause	2	5
Hypertensive disorders	1	2.5
Uncertain	1	2.5

**Table 3:** The mortality index of near-miss events in Kassala hospital, Sudan: 2008-2010

Near miss event	Number of women died	Number of event	Mortality index
Infection	14	49	22.2%
Hemorrhage	9	93	8.8%
Anemia	3	27	10%
Dystocia	2	18	10%
Hypertensive disorders	1	41	02.4%

While gestational age (34.8 ± 8 vs. 25 ± 6.6 weeks, *P *< 0.001) in near-miss cases was significantly higher than that in maternal death cases, there was no significant difference in age, parity, educational levels, residence and antenatal care coverage in the near-miss cases compared with maternal deaths, table 4.

**Table 4:** Comparison of near-miss cases and maternal deaths in Kassala hospital, Sudan: 2008-2010

Variable	Near-miss cases(*N *= 205)	Maternal Deaths(*N *= 40)	*P*
*The mean ± SD (95% CI) of *			
Age, years	25.5*± *6 (25.6-27.3)	28.2*±*5.7 (26.4-29.9)	0.5
Parity	3.0.1*± *9 (2.7-3.2)	2.6*± *1.8 (2.0-3.1)	0.7
Gestational age, weeks	34.8*± *8.0 (33.7-35.8)	25.0*± *6.6 (22.9-27.0)	<0.001
*Number (%, 95% CI) of *			
Rural residence	133(64.9,58.1-71.1)	26(65.0,49.3-78.5)	0.9
Illiteracy	110(53.7, 46.8-60.4)	17(42.5, 27.9-58.0)	0.4
No antenatal care	139(67.8, 61.1-73.9)	22(55.0, 39.5-69.8)	0.1

## Discussion

This is the first report of maternal near-miss in Sudan. Maternal near-miss and its mortality index reflect the quality of care provided by a health facility. The current study showed a total maternal near-miss and maternal mortality ratio of 22.1/1000 live births and 432/100 000 live births, respectively. The maternal near-miss ratio which this study describes (22.1 per 1000 live births) is within the wide range of ratios reported in studies from other developing countries which used similar criteria for near-miss definition (12.3 - 82.3 per 1000 deliveries) [10,11]. It might not be valid to compare our results with results in industrialized countries because of different selection criteria for near-miss cases. The reduction of the present high rates of near-miss cases may be achieved by improving the resources for managing severe morbidities e.g. admission to the intensive care unit and organ/system failure [12,13]. In this hospital (Kassala) cases were managed in specialized ward for high risk care which is close to the labor room and operating theater. Generally, less than 10% of near-miss cases in low resource settings receive intensive care [14,15]. Furthermore, criteria for admission to intensive care unit is variant and depends on the availability and capacity of intensive care unit and on the institutional guidelines for intensive care unit admission [1].

In the current study, there is a high total mortality index for near-miss cases (19.5%), which mean that for every maternal death there are 5 near miss-cases. This reflects a poor care and unacceptable high maternal mortality in this setting. Septicemia and hemorrhage were the major causes of death and maternal morbidity in this study. A high maternal mortality ratio (644/100,000 live births), with septicemia and hemorrhage as the main direct causes, has been previously reported in the same hospital [9]. This signifies a poor response of the system to modify these obstetric complications or perhaps substandard care where no audit has been performed. Most (42.9%) of the cases were referred from rural hospitals, which are managed by medical officers who are not well trained in emergency obstetric care. Therefore, training of these providers as well as system management in all its levels might improve and ultimately change these results.

In this setting, the health care providers were faced with a high percentage of life-threatening obstetric situations. Complications resulted in near-miss and maternal deaths with septicemia, dystocia and anemia, with a higher mortality index, which constitute an important and significant threat to the survival of pregnant women. Despite the high morbidity from hemorrhage and hypertensive disorders (40.8%, 18%, respectively) their mortality index was lower than that of the other events. This could be due to the availability of blood bank services and the introduction of magnesium sulfate as a preventive measure for these events. An increased level of care and effort are required to deal with near-miss events with a high mortality index, e.g., infection, dystocia and maternal anemia.

In the current study, lack of antenatal care services, illiteracy and rural residence might be predictors for maternal mortality since the majority of the near miss cases were illiterate, had no antenatal care and of rural residence.

One of the limitations of this study is weakness of retrospective method of data collection with respect to the quality of those records, the availability of all records, the coding of diagnosing and the correctness of diagnosis. For future studies, prospective collection of data using clear definition of near-miss and including audit of the care given is advised. By doing so and introducing of the audit cycle, the quality of the maternal care might be improved.

## Conclusion

There is a high frequency of maternal morbidity and mortality at the level of this facility. Therefore maternal health policy needs to be concerned not only with averting the loss of life, but also with preventing or ameliorating maternal-near miss events (hemorrhage, infections, hypertension and anemia) at all care levels including primary level.

## Competing interests

The authors declare that they have no competing interests.

## Authors' contributions

AAA, AKH and AO carried out the study and participated in the statistical analysis and procedures. GKA and IA coordinated and participated in the study design, statistical analysis and the drafting of the manuscript. All the authors read and approved the final version.

## Pre-publication history

The pre-publication history for this paper can be accessed here:

http://www.biomedcentral.com/1471-2393/11/48/prepub
